# Deep Learning Soft-Decision GNSS Multipath Detection and Mitigation

**DOI:** 10.3390/s24144663

**Published:** 2024-07-18

**Authors:** Fernando Nunes, Fernando Sousa

**Affiliations:** 1 Instituto de Telecomunicações, 1049-001 Lisboa, Portugal; fsousa@isel.pt; 2 Instituto Superior Técnico, Universidade de Lisboa, Torre Norte, Piso 10, 1049-001 Lisboa, Portugal; 3 Instituto Superior de Engenharia de Lisboa, Instituto Politécnico de Lisboa, 1959-007 Lisboa, Portugal

**Keywords:** multipath detection, multipath mitigation, deep learning, convolutional neural network, multilayer perceptron

## Abstract

A technique is proposed to detect the presence of the multipath effect in Global Navigation Satellite Signal (GNSS) signals using a convolutional neural network (CNN) as the building block. The network is trained and validated, for a wide range of $C/N_{0}$ values, with a realistic dataset constituted by the synthetic noisy outputs of a 2D grid of correlators associated with different Doppler frequencies and code delays (time-domain dataset). Multipath-disturbed signals are generated in agreement with the various scenarios encompassed by the adopted multipath model. It was found that pre-processing the outputs of the correlators grid with the two-dimensional Discrete Fourier Transform (frequency-domain dataset) enables the CNN to improve the accuracy relative to the time-domain dataset. Depending on the kind of CNN outputs, two strategies can then be devised to solve the equation of navigation: either remove the disturbed signal from the equation (hard decision) or process the pseudoranges with a weighted least-squares algorithm, where the entries of the weighting matrix are computed using the analog outputs of the neural network (soft decision).

## 1. Introduction

Multipath is one of the major sources of positioning errors in GNSS receivers operating near the Earth surface. It is due to the reception of one or more reflected rays besides (or instead of) the direct ray or line of sight (LOS). The reflected rays are characterized by extra delays relative to the arrival of the LOS signal. The reflected signals may also exhibit different Doppler frequencies, for instance when the receiver is not static. Multipath aspects affect both the code and carrier measurements, although the magnitudes of the errors differ significantly [1].

Many different methods have been proposed to mitigate the effect of multipath on the computation of the position, velocity, and time of the GNSS receiver at different stages, from the antenna to post-detection Receiver Autonomous Integrity Monitoring (RAIM) techniques [2,3]. An important class of mitigation techniques operate at the correlators level, including non-parametric and parametric techniques. Non-parametric processing, such as double-delta processing [4], resorts to code discriminator designs that are less sensitive to multipath-induced errors, while parametric processing, such as the Multipath Estimating Delay Lock Loop (MEDLL) [5], tries to estimate the parameters associated with the reflected rays. In any case, it will be beneficial if reliable side information, concerning the degradation of a given signal by multipath aspects, is available. In fact, the existence of a binary multipath/no multipath classifier permits the receiver to adopt one of the following alternatives. Strategy I: remove the signal affected by multipath from the navigation equation, provided that the number of visible satellites exceeds the minimum of 4. Strategy II: keep the multipath-disturbed signal in the navigation equation but process it in a different way from the other (unaffected) signals, using, for instance, a weighted least-squares estimator.

In the last two decades, several authors have applied machine learning (algorithms that can learn from experience) with various degrees of success to detect and mitigate multipath in GNSS receivers using supervised or unsupervised learning and different datasets [6,7,8,9,10,11,12,13]. References [6,10] use multilayer perceptrons, [8] utilizes a K-means clustering technique with unsupervised learning, and the remaining works resort to convolutional neural networks (CNNs). In [14], the authors replace standard correlation schemes with deep neural network-based correlation schemes to learn the complexity of the multipath channels. A technique based on the rain forest learning algorithm is proposed in [15] to estimate the multipath parameters and remove the estimated reflected signal components. A thorough survey on the application of machine learning techniques in different aspects of GNSS signal processing, including multipath mitigation, is provided in [16]. Reference [17] reviews the previous work on multipath mitigation using machine learning techniques, with the received signal strength, elevation angle, and receiver correlator outputs constituting the most popular input features.

Herein, we propose a multipath/no multipath classifier based on a CNN with supervised learning that uses synthetic signals disturbed by additive correlated noise during the training stage. The dataset is generated by a 2D grid of correlators with different code delays and Doppler frequencies. The existence of multipath provokes the change in the correlator outputs, and these features can be captured by the neural network. Since the dataset is 2D, the problem is similar to the feature extraction in images, where the CNNs have been immensely successful [18]. The adopted CNN is trained and validated with noisy synthetic signals for a wide range of carrier-to-noise ratios, namely $30\leq C/N_{0}\leq 50$ dB-Hz. The dataset is generated using a blend of different environments: open, rural, suburban, urban, and highway. These environments constitute the DLR (German Aerospace Center) satellite–ground model proposed in [19,20]. Alternatively, the multipath environment could have been characterized by the more complicated model proposed in [21], which is adopted, for instance, in [14].

Extensive simulations have shown that the best results are obtained when the CNN inputs are pre-processed with a two-dimensional Discrete Fourier Transform. The proposed algorithms are well-suited for software receivers, which employ an analog-to-digital converter that captures all the channels and demodulates the channel waveforms using software on a general-purpose processor [22]. Depending on the type of neural network outputs (hard or soft decisions), either strategy I or II may be applied to solve the equation of navigation.

The paper is organized as follows. In Section 2, we characterize the outputs of the grid of correlators in the presence of multipath and Gaussian additive noise. Section 3 describes the DLR multipath model used to train the networks and addresses the training of two competing neural networks: the multilayer perceptron (MLP) and the convolutional neural network (CNN), with the MLP serving as the benchmark. In Section 4, the performance of the selected solution (CNN), using hard and soft decisions, is evaluated and a multipath mitigation technique is proposed. Finally, conclusions are drawn in Section 5.

## 2. Correlators Characterization

**Figure 1:**
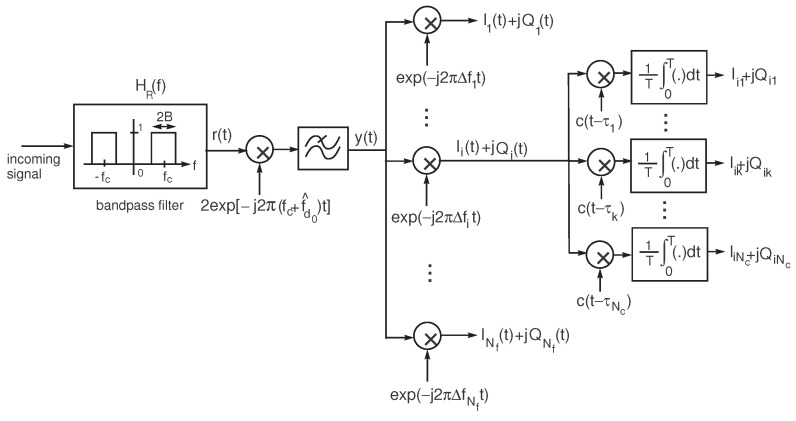
Grid of correlators.

Assume that the receiver includes a front-end that heterodynes the GNSS signal, r(t), transmitted by each satellite, to produce the complex baseband signal y(t) and a grid of $N_{f}N_{c}$ correlators, as sketched in Figure 1. In the scheme, 2B is the front-end bandwidth. The frequency $f_{c}+{\hat{f}}_{d_{0}}$ is derived from locking the local oscillator to the incoming signal, with $f_{c}$ denoting the nominal GNSS carrier frequency. The correlators are separated in frequency by $\Delta f=2f_{dop}/N_{f}$ and in the code delay by $\Delta \tau =2/N_{c}$ (in chip units). The maximum Doppler frequency range, $2f_{dop}$, should be adjusted for every scenario to encompass the different carrier frequencies of the multipath replicas that hit the receiver’s antenna.

Using a software receiver, the analog signal y(t) is sampled and the rest of the operations leading to the correlators output will be performed with digital signals (although this is not shown in Figure 1 for simplicity). The correlators outputs are provided by

(1) $$Z_{ik}=I_{ik}+jQ_{ik}=\frac{1}{T}\int_{0}^{T}y\left(t\right)\exp\left(-j2\pi \Delta f_{i}t\right)c\left(t-\tau_{k}\right)\;dt,$$

where *T* is the duration of the correlation interval and c(t) is a pseudorandom (PRN) code sequence with chip duration $T_{c}$ and |c(t)|=1. Conducting $\Delta f_{i}=f_{min}+i\Delta f$, $i=0,\ldots ,N_{f}-1$, we obtain

(2) $$Z_{ik}=\frac{1}{T}\int_{0}^{T}x_{k}\left(t\right)\exp\left(-j2\pi i\Delta ft\right)\;dt,$$

with $x_{k}\left(t\right)=y\left(t\right)\exp\left(-j2\pi f_{min}t\right)c\left(t-\tau_{k}\right)$. In software receivers, the quantities in (2) are calculated from the samples $x_{k}\left(t_{m}\right)$ as

(3) $$Z_{ik}\approx \frac{1}{M}\sum_{m=0}^{M-1}x_{k}\left(t_{m}\right)\exp\left(-j\frac{2\pi im\mu }{M}\right),$$

in which $t_{m}=m\Delta t$, where Δt=T/M is the sampling interval and μ=ΔfT.

Consider that, in the presence of multipath, the received GNSS signal r(t) is constituted by a strong ray and $N_{r}$ weaker rays plus additive Gaussian noise w(t). The noise in-band power spectrum is $G_{w}\left(f\right)=N_{0}/2$ for $f_{c}-B\leq \left|f\right|\leq f_{c}+B$. The bandpass signal is

(4) $$r\left(t\right)=\sum_{n=0}^{N_{r}}A_{n}D\left(t-\tau_{n}\right)c\left(t-\tau_{n}\right)\cos\left[2\pi \left(f_{c}+f_{d_{n}}\right)t+\theta_{n}\right]+w\left(t\right).$$

Each ray of index *n* is characterized by amplitude $A_{n}$, propagation delay $\tau_{n}$, Doppler frequency $f_{d_{n}}$, and phase $\theta_{n}$. D(t) is the navigation message, with |D(t)|=1. In the case of a pilot channel, the navigation message is constant with D(t)=1 (this condition will be assumed hereafter). The index n=0 corresponds to the strongest ray, which coincides, in general, with the line-of-sight (LOS) ray when it is not obstructed. The bandwidth 2B is considered sufficient to accommodate the incoming signals in the presence of Doppler frequency shifts. Signal r(t) is converted to baseband using the local oscillator complex signal $2\exp[-j2\pi \left(f_{c}+{\hat{f}}_{d_{0}}\right)t]$. For the sake of simplicity, it is assumed that the carrier tracking loop is perfectly synchronized with the strongest incoming signal; that is, ${\hat{f}}_{d_{0}}=f_{d_{0}}$. The result is

(5) $$y\left(t\right)=\sum_{n=0}^{N_{r}}A_{n}c\left(t-\tau_{n}\right)\exp\left[j\left(2\pi \left(f_{d_{n}}-f_{d_{0}}\right)t+\theta_{n}\right)\right]+N\left(t\right),$$

where N(t)=NI(t)+jNQ(t), with NI(t) and NQ(t) being independent, zero mean, inphase/quadrature components of w(t) with equal powers $2N_{0}B$. The baseband signal y(t) is then multiplied by a grid of $N_{f}N_{c}$ complex units, $\exp\left(-j2\pi \Delta f_{i}t\right)\cdot c\left(t-\tau_{k}\right)$, $i=1,\ldots ,N_{f}$, $k=1,\ldots ,N_{c}$, to yield a 2D correlation, as sketched in Figure 1.

For the signal model described in (4), the correlators outputs are

(6) $$\begin{array}{ccc} Z_{ik}\; & = & \;\frac{1}{T}\int_{0}^{T}\left[I_{i}\left(t\right)+jQ_{i}\left(t\right)\right]c\left(t-\tau_{k}\right)\;dt\\ & = & \;\sum_{n=0}^{N_{r}}A_{n}\exp\left(j\theta_{n}\right)\frac{1}{T}\int_{0}^{T}\exp\left[j2\pi \left(f_{d_{n}}-f_{d_{0}}-\Delta f_{i}\right)t\right]c\left(t-\tau_{n}\right)c\left(t-\tau_{k}\right)\;dt,\\ & + & \;N_{ik}, \end{array}$$

with the noise component being

(7) $$N_{ik}={NI}_{ik}+j{N\;Q}_{ik}=\frac{1}{T}\int_{0}^{T}N\left(t\right)\exp\left(-j2\pi \Delta f_{i}t\right)c\left(t-\tau_{k}\right)\;dt.$$

The integrals in (6) are of the form

(8) $$I_{c}=\frac{1}{T}\int_{0}^{T}c\left(t-\tau_{n}\right)c\left(t-\tau_{k}\right)\exp\left(j2\pi \varphi t\right)\;dt.$$

Assuming that the signal exp(j2πφt) is slowly varying in each interval of duration $T_{p}=T/P$, with P≫1, we may write

(9) $$I_{c}\approx \frac{1}{P}\sum_{p=1}^{P}\exp\left[j2\pi \varphi \left(p-1/2\right)T_{p}\right]\frac{1}{T_{p}}\int_{\left(p-1\right)T_{p}}^{pT_{p}}c\left(t-\tau_{n}\right)c\left(t-\tau_{k}\right)\;dt.$$

Let $R_{c}\left(\tau \right)$ denote the autocorrelation function of code c(t) with period $ST_{c}$

(10) $$R_{c}\left(\tau \right)=\frac{1}{ST_{c}}\int_{0}^{ST_{c}}c\left(t\right)c\left(t-\tau \right)\;dt\approx \frac{1}{T_{p}}\int_{\left(p-1\right)T_{p}}^{pT_{p}}c\left(t\right)c\left(t-\tau \right)\;dt,$$

and $T_{p}<ST_{c}$, leading to

(11) $$\begin{array}{ccc} I_{c}\; & \approx & \;\frac{1}{P}R_{c}\left(\tau_{k}-\tau_{n}\right)\sum_{p=1}^{P}\exp\left[j2\pi \varphi \left(p-1/2\right)T_{p}\right]\\ & = & \;\frac{1}{P}R_{c}\left(\tau_{k}-\tau_{n}\right)\exp\left(j\pi \varphi T\right)\frac{\sin(\pi \varphi T)}{\sin(\pi \varphi T_{p})}. \end{array}$$

For $|\varphi T_{p}|\leq 0.2$, the Dirichlet kernel $\sin\left(\pi \varphi PT_{p}\right)/\left[P\sin\left(\pi \varphi T_{p}\right)\right]$, is well-approximated by the sinc function, defined as sinc(x)≡sin(πx)/(πx), yielding $I_{c}\approx R_{c}\left(\tau_{k}-\tau_{n}\right)\mathrm{sinc}\left(\varphi T\right)$exp(jπφT). Thus, (6) can be written approximately as

(12) $$\begin{array}{ccc} Z_{ik}\; & \approx & \;\sum_{n=0}^{N_{r}}A_{n}R_{c}\left(\tau_{k}-\tau_{n}\right)\;\mathrm{sinc}\left[\left(f_{d_{n}}-f_{d_{0}}-\Delta f_{i}\right)T\right]\exp\left\{j\left[\pi \left(f_{d_{n}}-f_{d_{0}}-\Delta f_{i}\right)T+\theta_{n}\right]\right\}\\ & + & \;N_{ik}. \end{array}$$

The cross-correlation of the noise components of two correlators, $N_{ik}$ and $N_{lm}$, is defined as

(13) $$\begin{array}{ccc} & & E\{N_{ik}N_{lm}^{*}\}=\\ & & \frac{1}{T^{2}}\int_{0}^{T}\int_{0}^{T}E\left\{N\left(t\right)N^{*}\left(\lambda \right)\right\}\exp\left[-j2\pi \left(\Delta f_{i}t-\Delta f_{l}\lambda \right)\right]c\left(t-\tau_{k}\right)c\left(\lambda -\tau_{m}\right)\;dt\;d\lambda . \end{array}$$

The determination of the cross-correlation is complicated except when BT≫1. In this case, $E\left\{N\left(t\right)N^{*}\left(\lambda \right)\right\}\approx 2N_{0}\delta \left(t-\lambda \right)$ and

(14) $$\begin{array}{ccc} & & E\{N_{ik}N_{lm}^{*}\}\approx \\ & & \frac{2N_{0}}{PT}\sum_{p=1}^{P}\exp\left[-j2\pi \left(\Delta f_{i}-\Delta f_{l}\right)\left(p-1/2\right)T_{p}\right]\frac{1}{T_{p}}\int_{\left(p-1\right)T_{p}}^{pT_{p}}c\left(\lambda -\tau_{k}\right)c\left(\lambda -\tau_{m}\right)\;d\lambda . \end{array}$$

Therefore,

(15) $$E\left\{N_{ik}N_{lm}^{*}\right\}\approx \frac{2N_{0}}{PT}R_{c}\left(\tau_{m}-\tau_{k}\right)\exp\left[j\pi \left(\Delta f_{l}-\Delta f_{i}\right)T\right]\frac{\sin[\pi \left(\Delta f_{l}-\Delta f_{i}\right)T]}{\sin[\pi \left(\Delta f_{l}-\Delta f_{i}\right)T_{p}]}$$

and, assuming that $|\left(\Delta f_{l}-\Delta f_{i}\right)T_{p}|\ll 1$, we have

(16) $$E\left\{N_{ik}N_{lm}^{*}\right\}\approx \frac{2N_{0}}{T}R_{c}\left(\tau_{m}-\tau_{k}\right)\exp\left[j\pi \left(\Delta f_{l}-\Delta f_{i}\right)T\right]\;\mathrm{sinc}\left[\left(\Delta f_{l}-\Delta f_{i}\right)T\right].$$

Taking into account (7) and (16), we obtain the following expressions for the real-valued cross-correlations

(17) $$\begin{array}{ccc} \left[\begin{array}{c} E\{{NI}_{ik}{NI}_{lm}\}\\ E\{{NQ}_{ik}{NI}_{lm}\} \end{array}\right]\; & = & \;\left[\begin{array}{c} E\{{NQ}_{ik}{NQ}_{lm}\}\\ -E\{{NI}_{ik}{NQ}_{lm}\} \end{array}\right]\\ & \approx & \;\frac{N_{0}}{T}R_{c}\left(\tau_{m}-\tau_{k}\right)\;\mathrm{sinc}\left[\left(\Delta f_{l}-\Delta f_{i}\right)T\right]\left[\begin{array}{c} \cos[\pi \left(\Delta f_{l}-\Delta f_{i}\right)T]\\ \sin[\pi \left(\Delta f_{l}-\Delta f_{i}\right)T] \end{array}\right]. \end{array}$$

Using Monte Carlo simulation, the noise random variables (r.v.) ${NI}_{ik}$ and ${NQ}_{ik}$ have to be generated for all the elements of the grid $i=1,\cdots ,N_{f},\;k=1,\cdots ,N_{c}$. To this end, we concatenate the noise matrices $NI(N_{f}\times N_{c})$ and $NQ(N_{f}\times N_{c})$ into a single vector U(p), $p=1,\ldots ,2N_{f}N_{c}$, according to $U\left(\left(i-1\right)N_{c}+k\right)=NI_{ik}$ and $U\left(\left(i-1\right)N_{c}+k+N_{f}N_{c}\right)=NQ_{ik}$.

The covariance matrix $C=E\{UU^{T}\}$ of the resulting vector $U(2N_{f}N_{c}\times 1)$ has $4N_{f}^{2}N_{c}^{2}$ elements. For $p,q=1,\ldots ,2N_{f}N_{c}$ and p≤q, each element of C is provided by

(18) $$C_{pq}=E\left\{U\left(p\right)U\left(q\right)\right\}=\left\{\begin{array}{cc} E\{{NI}_{ik}{NI}_{lm}\}, & q\leq N_{f}N_{c}\;(\mathrm{case}\;I)\\ E\{{NI}_{ik}{NQ}_{lm}\}, & p\leq N_{f}N_{c},\;q>N_{f}N_{c}\;(\mathrm{case}\;\mathrm{II}),\\ E\{{NQ}_{ik}{NQ}_{lm}\}, & p>N_{f}N_{c}\;(\mathrm{case}\;\mathrm{III}) \end{array}\right.$$

with $C_{qp}=C_{pq}$. For the three cases, the indices of the elements of C are computed as

Case I: $p=\left(i-1\right)N_{c}+k,\;q=\left(l-1\right)N_{c}+m$.

Case II: $p=\left(i-1\right)N_{c}+k,\;q=\left(l-1\right)N_{c}+m+N_{f}N_{c}$.

Case III: $p=\left(i-1\right)N_{c}+k+N_{f}N_{c},\;q=\left(l-1\right)N_{c}+m+N_{f}N_{c}$.

After the covariance matrix is determined, *U* can be easily generated from vector $W(2N_{f}N_{c}\times 1)$ of Gaussian, zero-mean, unity power, and independent components using the technique described in Appendix B.

## 3. Neural Network Training

### 3.1. Multipath Model

In order to train the neural network (NN), we assume a certain ensemble of models to characterize multipath. This does not imply that the experimental multipath to be detected obeys those models. In fact, in practice, it is almost impossible to assign a set of models that encompasses all cases encountered of real multipath. Nevertheless, the NN should be trained with the largest possible set of plausible multipath models.

Land mobile satellite (LMS) channels are usually divided into narrowband and wideband models. While the narrowband models describe the channel by a multiplicative operation on the signal, the wideband models take into account the frequency dependency caused by the signal echoes [23]. Echoes with different delays can be resolved when the difference in delay is larger than the inverse of the corresponding receiver (baseband) bandwidth: $\tau_{m}-\tau_{k}>1/B$. For instance, the receiver reference bandwidth of Galileo signal E1 is 2B=24.552 MHz [24], which corresponds to a minimum resolvable difference of delays of approximately 81.5 ns (approximately 24.5 m).

We consider, henceforth, for the purpose of NN training, a wideband model of LMS communications affected by multipath fading and signal shadowing. The $N_{r}$ different reflectors cause echoes with delays $\tau_{m}\left(t\right)=\tau_{0}\left(t\right)+\Delta \tau_{m}\left(t\right)$, where $\tau_{0}\left(t\right)$ is the propagation delay of the direct ray and $\Delta \tau_{m}\left(t\right)$ is the excess delay of each reflected ray. A simple but efficient model for wideband LMS channels is the tapped delay line model, where each tap is described by a narrowband model. Assume that the transmitted signal is represented as s(t)=Re{sbb(t)ej2πfct}, where $s_{bb}\left(t\right)$ is the corresponding complex envelope. In the case of multiple propagation paths, the equivalent lowpass channel is described by the time-variant impulse response [25]

(19) $$h\left(\tau ;t\right)=\sum_{m=0}^{N_{r}}E_{m}\left(t\right)\delta \left(\tau -\tau_{m}\left(t\right)\right),\;E_{m}\left(t\right)=\alpha_{m}\left(t\right)e^{-j2\pi f_{c}\tau_{m}\left(t\right)}.$$

Let the satellite-receiver distance traveled by the mth path, in a short time interval, change approximately linearly with time (constant Doppler frequency). The channel impulse response may be written as

(20) $$h\left(\tau ;t\right)=\sum_{m=0}^{N_{r}}\alpha_{m}\left(t\right)\exp\left[j\left(2\pi f_{m}t-\phi_{m}\right)\right]\delta \left(\tau -\tau_{m}\left(t\right)\right),$$

which means that each echo is characterized by the following quantities: $\alpha_{m}$ (amplitude), $f_{m}$ (Doppler frequency), $\phi_{m}$ (phase), and $\tau_{m}$ (delay).

**Figure 2:**
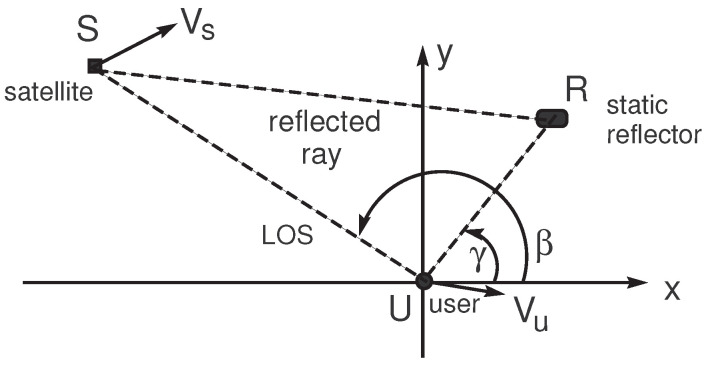
Multipath scenario.

Admit that the receiver’s phase lock loop is synchronized to the carrier frequency of the direct ray (LOS), which includes the component due to the Doppler effect. Next, we characterize the Doppler frequency deviation $\Delta f_{m}=f_{m}-f_{0}$ for each reflected ray relative to the LOS. We follow the simplified scenario where the propagation between the satellite and the user (receiver) is constituted by an LOS and a reflected ray, as described in Figure 2. The reflector is considered static.

If the coordinates of the satellite, reflector, and receiver are, respectively, $\left(x_{s}\left(t\right),y_{s}\left(t\right)\right)$, $\left(x_{r},y_{r}\right)$, and $\left(x_{u}\left(t\right),y_{u}\left(t\right)\right)$, the lengths of the direct ray (LOS) and the reflected ray will be provided by

(21) $$L_{0}\left(t\right)=\sqrt{{\left[x_{u}\left(t\right)-x_{s}\left(t\right)\right]}^{2}+{\left[y_{u}\left(t\right)-y_{s}\left(t\right)\right]}^{2}}$$

and

(22) $$L_{1}\left(t\right)+L_{2}\left(t\right)=\sqrt{{\left[x_{r}-x_{s}\left(t\right)\right]}^{2}+{\left[y_{r}-y_{s}\left(t\right)\right]}^{2}}+\sqrt{{\left[x_{u}\left(t\right)-x_{r}\right]}^{2}+{\left[y_{u}\left(t\right)-y_{r}\right]}^{2}}.$$

The satellite–user velocity using the LOS is

(23) $$V_{0}\left(t\right)=\frac{dL_{0}\left(t\right)}{dt}=\frac{1}{L_{0}\left(t\right)}\left(\left[x_{u}\left(t\right)-x_{s}\left(t\right)\right]\left(V_{ux}-V_{sx}\right)+\left[y_{u}\left(t\right)-y_{s}\left(t\right)\right]\left(V_{uy}-V_{sy}\right)\right),$$

where the satellite and user velocity vectors are, respectively, $V_{s}=\left(V_{sx},V_{sy}\right)$ and $V_{u}=\left(V_{ux},V_{uy}\right)$. The apparent satellite–user velocity using the reflected ray is

(24) $$\begin{array}{ccc} V_{12}\left(t\right)\; & = & \;\frac{dL_{1}\left(t\right)}{dt}+\frac{dL_{2}\left(t\right)}{dt}\\ & = & \;-\frac{1}{L_{1}\left(t\right)}\left(\left[x_{r}-x_{s}\left(t\right)\right]V_{sx}+\left[y_{r}-y_{s}\left(t\right)\right]V_{sy})\right)+\\ & & \;\frac{1}{L_{2}\left(t\right)}\left(\left[x_{u}\left(t\right)-x_{r}\right]V_{ux}+\left[y_{u}\left(t\right)-y_{r}\right]V_{uy}\right). \end{array}$$

Since, in general, $L_{1}\left(t\right)\approx L_{0}\left(t\right)$ and $L_{2}\left(t\right)\ll L_{0}\left(t\right)$, we have $V_{12}\left(t\right)-V_{0}\left(t\right)\approx V_{ux}\left(\cos\beta -\cos\gamma \right)+V_{uy}\left(\sin\beta -\sin\gamma \right)$. Consider now, without loss of generality, that the receiver travels along the X-axis. Then, $V_{uy}=0$, and we obtain for the Doppler frequency deviation

(25) $$\Delta f_{d}=-\frac{V_{12}-V_{0}}{c}f_{c}=\frac{V_{ux}\left(\cos\gamma -\cos\beta \right)}{c}f_{c}.$$

The maximum absolute value of the Doppler frequency deviations is equal to $2|V_{ux}|f_{c}/c$, corresponding for instance to β=π and γ=0. Namely, if the receiver is traveling at the speed of 140 km/h (typical car speed on a highway) and $f_{c}=1.57$ GHz, the maximum absolute value will be ≈400 Hz (Doppler frequency range equal to ±400 Hz).

The DLR model for the LMS channel, herein adopted, divides the channel impulse response with $N_{r}+1$ rays into three parts [19,20]:

1. Direct path. There are two states for the direct ray: shadowing [bad channel state] and LOS (no shadowing) [good channel state]. The probability of each state depends on the type of environment: open, rural, suburban, urban, and highway (see Table A1 in Appendix A). For LOS conditions, a Rice distribution describes the probability density function (pdf) of the signal amplitude
  (26) $$p_{\alpha_{0}}\left(x\right)=\frac{x}{\sigma^{2}}I_{0}\left(\frac{x}{\sigma^{2}}\right)\exp\left(-\frac{x^{2}+1}{2\sigma^{2}}\right),\;x\geq 0,$$
  where $I_{0}\left(\cdot \right)$ is the zeroth-order modified Bessel function of the first kind and the Rice factor $\rho =1/(2\sigma^{2})$ denotes the carrier-to-multipath ratio. The corresponding cumulative distribution function (CDF) is [25]
  (27) $$F_{\alpha_{0}}\left(x\right)=1-Q\left(\frac{1}{\sigma },\frac{x}{\sigma }\right),\;x\geq 0,$$
  where Q(·,·) is Marcum’s Q function.
  In shadowed environments (bad channel state), $\alpha_{0}$ is Rayleigh-distributed with lognormal-distributed mean power $P_{0}=2\sigma^{2}$. The result is the Suzuki distribution [26]. That is,
  (28) $$\begin{array}{ccc} & & p_{\alpha_{0}}\left(x\right)=\frac{x}{\sigma^{2}}\exp\left(-\frac{x^{2}}{2\sigma^{2}}\right),\;x\geq 0, \end{array}$$
  (29) $$\begin{array}{ccc} & & p_{P_{0}}\left(x\right)=\frac{10}{\sqrt{2\pi }\sigma \ln10}\frac{1}{x}\exp\left[-\frac{{\left(10\log_{10}x-\mu \right)}^{2}}{2\sigma^{2}}\right],\;x>0, \end{array}$$
  where μ is the mean power level decrease in dB and $\sigma^{2}$ is the variance of the power level expressed in dB due to shadowing. The dimension of $\sigma^{2}$ is ${\mathrm{dB}}^{2}$.
  The CDF of $p_{\alpha_{0}}\left(x\right)$ is $F_{\alpha_{0}}\left(x\right)=1-\exp\left[-x^{2}/\left(2\sigma^{2}\right)\right],\;\;x\geq 0$. The CDF of $p_{P_{0}}\left(x\right)$ is
  (30) $$F_{P_{0}}\left(x\right)=\frac{1}{2}\left[1+\mathrm{erf}\left(\frac{10\log_{10}x-\mu }{\sigma \sqrt{2}}\right)\right],\;x>0,$$
  with the error function being defined by
  (31) $$\mathrm{erf}\left(z\right)=\frac{2}{\sqrt{\pi }}\int_{0}^{z}\exp\left(-t^{2}\right)\;dt.$$
2. Near echoes. A number of near echoes appear in the close vicinity of the receiver, with excess delays not exceeding $\tau_{e}=600$ ns. Most of the echoes will occur in this delay interval. The mean power of near echoes $S\left(\tau \right)=E\left\{\alpha_{m}^{2}\right\}$ is exponentially decreasing: $S\left(\tau \right)=S_{0}\exp\left(-\mu \tau \right)$. Given a mean echo power S(τ) for a fixed delay τ, the amplitude $\alpha_{m}^{\left(n\right)}$ of the near echoes will vary around this mean value according to a Rayleigh distribution with $2\sigma^{2}=S\left(\tau \right)$. The number of near echoes is Poisson-distributed, with mean λ. Recall that the Poisson distribution provides the probability that a certain number of independent events occur in a given interval (of time or space) when, on average, λ events occur in that interval [27]. The corresponding pdf is
  (32) $$p\left(x\right)=e^{-\lambda }\sum_{n=0}^{\infty }\frac{\lambda^{n}}{n!}\delta \left(x-n\right)$$
  and the CDF is
  (33) $$F\left(x\right)=e^{-\lambda }\sum_{n=0}^{\left\lfloor x\right\rfloor }\frac{\lambda^{n}}{n!},$$
  with ⌊x⌋ denoting the largest integer not exceeding *x*. The delay distribution $\Delta {\tau_{m}}^{\left(m\right)}$ of the near echoes follows an exponential distribution with pdf $p_{\Delta \tau_{n}}\left(x\right)=b^{-1}\exp\left(-x/b\right)$ and corresponding CDF $F_{\Delta \tau_{n}}\left(x\right)=1-\exp\left(-x/b\right)$, x≥0.
  The mean power of the near echoes $S\left(\tau \right)=E\left\{a_{k}^{2}\right\}$ is exponentially decreasing with the delay $S\left(\tau \right)=S_{0}\exp\left(-\nu \tau \right)$, ν>0, or in logarithmic scale $S_{dB}\left(\tau \right)=S_{0,dB}-d\tau_{\mu s}$, d>0, with *d* being expressed in dB/μs.
  For the adopted parameters of the near echoes, see Table A2.
3. Far echoes. The number of far echoes is Poisson-distributed. The far echoes appear with delays $\tau_{e}<\Delta \tau_{m}\leq \tau_{\max}$. The amplitudes $\alpha_{m}^{\left(f\right)}$ of the far echoes follow a Rayleigh distribution. The delays $\Delta {\tau_{m}}^{\left(f\right)}$ of the far echoes are uniformly distributed in [$\tau_{e},\tau_{\max}$].
  The adopted parameters of the far echoes are indicated in Table A3.

The direct ray is affected by obstacles, such as trees, whereas the echoes are affected by the presence of reflectors (buildings, mountains, etc.). The set of parameters are distinguished by the environments: the near echoes are determined by the foreground environment and the far echoes are determined by the background environment.

### 3.2. NN Characterization

In this work, we assume supervised learning for the NN, which relies on learning from a dataset with labels for each of the examples. There are two types of supervised learning: classification and regression [28]. Classification is used to determine the class that the data belong to and regression extracts a real value from the data. We consider the application of two different NN types to the problem of detecting GNSS signals affected by multipath aspects: a multilayer perceptron (MLP) and a convolutional NN (CNN). CNNs have been extensively applied in different fields, including computer vision, speech processing, face recognition, etc. [18]. Unlike the MLPs, which are conventional fully connected networks, shared weights and local connections are employed in the CNNs to make full use of 2D input-data structures, like image signals. This operation utilizes an extremely small number of parameters, which both simplifies the training process and speeds up the network [29].

For both NNs, we have considered the following alternative types of inputs: (mode 1), the correlators complex outputs $Z_{ik}$, displayed in Figure 1 [ time-domain inputs], or (mode 2), the corresponding Discrete Fourier Transform (DFT) [frequency-domain inputs], which are defined as

(34) $$Y_{rs}=\sum_{i=0}^{N_{f}-1}\exp\left(-j\frac{2\pi ir}{N_{f}}\right)\sum_{k=0}^{N_{c}-1}Z_{ik}\exp\left(-j\frac{2\pi ks}{N_{c}}\right),$$

with $r=0,\ldots ,N_{f}-1$ and $s=0,\ldots ,N_{c}-1$.

The use of the two types of NN inputs was motivated by the fact that, although signal processing concerning multipath detection and mitigation is predominantly carried out in the time domain, some research has also been performed in the frequency domain [30].

**Figure 3:**
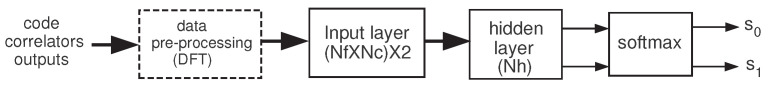
Block diagram of the MLP.

**Figure 4:**
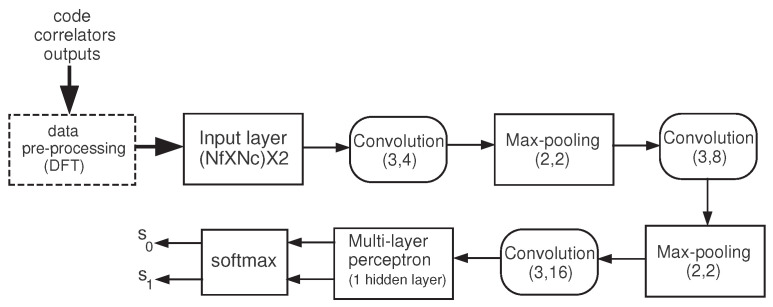
Block diagram of the CNN.

For the MLP, we have considered the structure depicted in Figure 3, which contains a single hidden layer with $N_{h}$ neurons. In contrast, the architecture of the adopted CNN consists of two main parts: feature extractors and a classifier. In the feature extraction layers, each layer of the network receives the output from its immediate previous layer as its input and passes its output to the input of the next layer. CNNs are built by repeatedly concatenating three classes of layers: convolutional, activation, and pooling. This structure is followed by a last stage that contains three fully connected layers and a classification layer [18,31]. The block diagram is shown in Figure 4. In the convolution block, the pair (a,b) indicates the filter size (a×a) and the number of filters (b). In the max-pooling block, the pair (c,d) indicates the pool size (c×c) and the 2D stride (d×d), where *d* is the common horizontal and vertical step size for traversing input. The NNs were implemented using Matlab, version R2022a.

The convolutional layer performs feature extraction by convolving the input with filters (kernels). After each convolution layer, a nonlinear activation layer is applied. We used the ReLU activation function f(x)=max{0,x}. The (sub-sampling) pooling layer performs nonlinear downsampling operations, which aims at reducing the spatial size of the representation while simultaneously decreasing the number of parameters, the possibility of overfitting, and the computational complexity of the network. The max-pooling function is used. In the last layer of the fully connected network, the softmax activation function is applied.

The NN is used as a binary classifier with classes $H_{0}$ (no multipath) and $H_{1}$ (multipath). In binary classification, the information about the success of a model is conveniently described by the confusion matrix, which contains four elements: true negative (TN), true positive (TP), false positive (FP), and false negative (FN) decisions. Accuracy is an informative measure of success, being defined as 1−Prob{decisionerror}, or

(35) $$\mathrm{accuracy}=1-(P_{fa}\cdot \mathrm{Prob}\left\{H_{0}\right\}+P_{md}\cdot \mathrm{Prob}\left\{H_{1}\right\}),$$

with $P_{fa}$ and $P_{md}$ denoting, respectively, the probabilities of false alarm and missed detection (or miss rate) (see, for instance, [32]). Thus,

(36) $$\begin{array}{ccc} & & P_{fa}=\mathrm{Prob}\left\{D_{1}|H_{0}\right\}=\frac{FP}{TN+FP},\\ & & P_{md}=\mathrm{Prob}\left\{D_{0}|H_{1}\right\}=\frac{FN}{TP+FN}, \end{array}$$

where $D_{0}$ and $D_{1}$ stand for decision in favor of classes $H_{0}$ and $H_{1}$, respectively. Additionally,

(37) $$\begin{array}{ccc} & & \mathrm{Prob}\left\{H_{0}\right\}=\frac{TN+FP}{TN+FP+TP+FN},\\ & & \mathrm{Prob}\left\{H_{1}\right\}=1-\mathrm{Prob}\left\{H_{0}\right\}=\frac{TP+FN}{TN+FP+TP+FN}. \end{array}$$

Previous formulas lead to [28,29]

(38) $$\mathrm{accuracy}=\frac{TN+TP}{TP+FN+FP+TN},$$

which is the evaluation metric we are going to use throughout this work. Other common metrics, different from accuracy, are the precision and the recall [28,29]. Precision is the fraction of multipath detections ($D_{1}$) that are correct, while recall is the fraction of multipath events that were detected. That is, precision is P=TP/(TP+FP) and recall is R=TP/(TP+FN), with $P_{md}=1-R$.

Equation (35) shows that, when the data are unbalanced, accuracy becomes biased towards the majority class and provides a wrong estimate of the decision success. In fact, when $\mathrm{Prob}\left\{P_{0}\right\}\gg \mathrm{Prob}\left\{P_{1}\right\}$, the accuracy will be almost independent of $P_{md}$, and, when $\mathrm{Prob}\left\{P_{0}\right\}\ll \mathrm{Prob}\left\{P_{1}\right\}$, the accuracy will be essentially insensitive to $P_{fa}$. In those cases, metrics such as the F1 score, defined as F1=2R·P/(R+P), provide more realistic results [28].

Consider that the output of the NN is the vector $O=(O_{0},\;O_{1})$. The hard tentative decision generated by the NN is ${\arg\;\max}_{k}O_{k}$. Alternatively, we can normalize the network outputs using the softmax function

(39) $$s_{k}=\frac{\exp(O_{k})}{\exp\left(O_{0}\right)+\exp\left(O_{1}\right)},\;k=0,1,$$

to compute the probability of each class. The softmax function maps the real-value network output to a probability distribution over a number of classes, where the number of classes equals the number of neurons in the final layer [28]. Notice that $s_{0}+s_{1}=1$.

## 4. Simulation Results

### 4.1. Single Observation Decisions

Before training the NN, we establish the maximum expected value of the Doppler frequency deviation of the reflected rays due to the multipath effect for the scenario under analysis. This value depends on the dynamics of the user (receiver) and the possible motion of the reflectors. Following the example referred to in Section 3 (typical car speed in a highway), we set the maximum absolute Doppler frequency deviation used by the grid of correlators to $f_{\mathrm{maxtrain}}=400$ Hz (or Doppler frequency range of ±400 Hz). The resulting frequency step in the bank of heterodyning units of Figure 1 is then $\Delta f=2f_{\mathrm{maxtrain}}/N_{f}=800/N_{f}$. Taking into account that multipath delayed signals only affect the receiver’s performance approximately in a chip duration interval [1], the delay step used by the bank of correlators is made equal to $\Delta \tau =2T_{c}/N_{c}$.

Both NNs are trained with 2D data formatted as $\left(N_{f}\times N_{c}\right)$ complex matrices, and 100,000 matrices were generated: 80% of the matrices are used to train the network and the remaining 20% are included in the validation set, whose goal is to determine whether the trained model is overfitting. For training and validation purposes, the data are generated assuming equal probabilities for hypotheses $H_{0}$ (multipath absent) and $H_{1}$ (multipath present). In case $H_{1}$, the data were generated assuming each of the near echo scenarios, open, rural, suburban, urban, and highway, with equal probabilities. The data are produced with different values of $C/N_{0}$ in the interval $30\leq C/N_{0}\leq 50$ dB-Hz, with a uniform distribution in dB-Hz units and correlation interval T=10 ms. In all the following results, the BOCs(1,1) modulation will be utilized. The use of modulation BPSK(1) as an alternative has revealed only minor differences in terms of the achieved accuracy. A learning rate of $5\times 10^{-4}$ was used with both types on NNs. To achieve the best performance, the range of Doppler frequency deviations, $\pm f_{\mathrm{maxtest}}$, used by the NN in test mode should verify $f_{\mathrm{maxtest}}\leq f_{\mathrm{maxtrain}}$.

The two NN architectures are tested with correlations outputs generated according to (12), with a maximum number of reflected rays $N_{r}=5$, assuming multipath and no-multipath scenarios, with equal probabilities. This value of $N_{r}$ is considered to be a reasonable amount of reflected rays in most multipath environments. In fact, simulations carried out with larger values of $N_{r}$ have revealed no significant differences in the NN behavior.

The amplitudes of the different rays are $A_{0}=1$ and $A_{k}$ ($k=1,\ldots ,N_{r}$) following a uniform distribution with $0<A_{k}<1$. The phases $\theta_{k}$ are independent and uniformly distributed in the interval $0\leq \theta_{k}<2\pi$. The delays $\tau_{k}$ are independent and uniformly distributed in the interval $0<\tau_{k}<T_{c}$. The Doppler frequency shifts $f_{d_{k}}-f_{d_{0}}$ are independent and uniformly distributed, such that $|f_{d_{k}}-f_{d_{0}}|<f_{\mathrm{maxtest}}$. The characterization of amplitudes, phases, delays, and Doppler frequencies as independent uniform random variables, although simplistic, seems to be the natural distribution choice, meaning that we have no a priori statistical knowledge of those variables. It is expected that, with real signals, the NNs have the capacity to withstand the possible non-uniformity of the variables.

**Figure 5:**
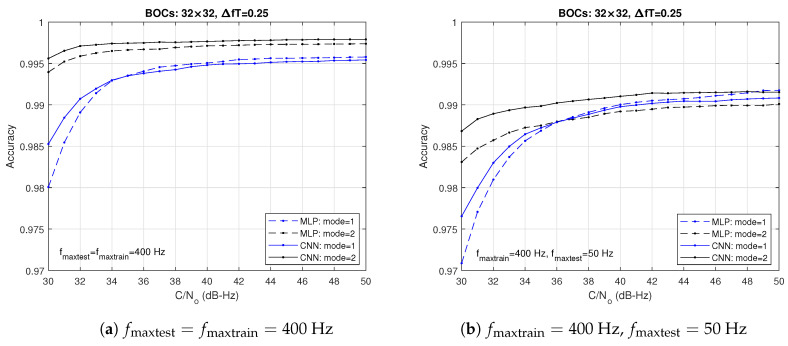
Accuracies obtained when time-domain (blue lines) and frequency-domain inputs (black lines) are used with a multilayer perceptron (MLP) and a convolutional neural network (CNN).

Figure 5 compares the accuracies achieved with the MLP and the CNN architectures when time-domain [mode 1] and frequency-domain [mode 2] data are used. The data are formatted as $\left(N_{f}\times N_{c}\right)=\left(32\times 32\right)$ matrices. In this simulation, we have considered two matching conditions for the Doppler frequencies: (a) $f_{\mathrm{maxtest}}=f_{\mathrm{maxtrain}}=400$ Hz (matching condition) and (b) $f_{\mathrm{maxtest}}=50$ Hz, $f_{\mathrm{maxtrain}}=400$ Hz (mismatch condition). The tests were performed with 40,000 data matrices. An MLP with one hidden layer constituted by $N_{h}=25$ neurons is employed in (a) and a convolutional neural NN (CNN) is applied in (b). The number of neurons indicated for the hidden layer of the MLP was found experimentally and is a trade-off between the accuracy achieved by the classifier and the complexity of the algorithm.

Comparing the two sets of plots, we can see that, overall, the results improve for large values of $\left(C/N_{0}\right)$, as expected, and there is a performance degradation of both NNs in the case of frequency mismatch. In all the cases, the CNN permits to obtain better results than the MLP, with the best performance being achieved with mode-2 data (black solid curves). It can be shown that this trend is essentially kept even if the values of $f_{\mathrm{maxtrain}}$ and $f_{\mathrm{maxtest}}$ change. The advantage of the CNN architecture is due to its double role as a feature extractor and classifier. Thus, in order to simplify the analysis, we will henceforth consider only the CNN architecture and mode-2 input data.

In Figure 5b, the NNs were not conveniently trained to match the Doppler frequency range of the test dataset. So, to avoid performance degradation, it is convenient to determine the approximate limits of the Doppler frequency deviation corresponding to each scenario, taking into account the range of the receiver speeds, and use a trained network as matched as possible to that expected range of Doppler frequency deviations.

**Figure 6:**
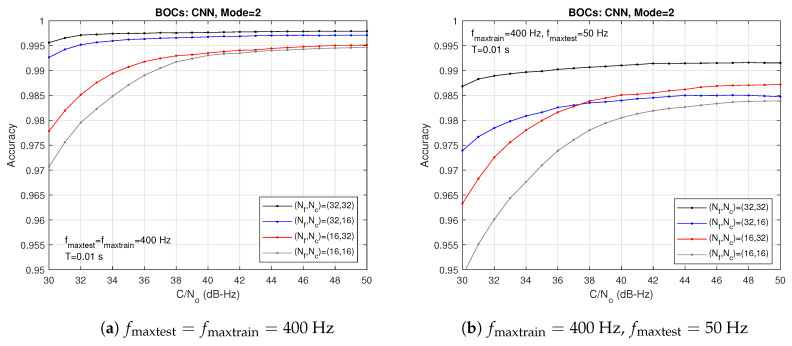
Accuracies obtained with correlators grids of different sizes $\left(N_{f},N_{c}\right)$ with $f_{\mathrm{maxtrain}}=400$ Hz.

Figure 6 exhibits the accuracies provided by correlators grids of different sizes $\left(N_{f}\times N_{c}\right)$. The NN was trained with $f_{\mathrm{maxtrain}}=400$ Hz. Two cases are considered: (a) matching case with $f_{\mathrm{maxtest}}=f_{\mathrm{maxtrain}}$ and (b) mismatch case with $f_{\mathrm{maxtest}}=f_{\mathrm{maxtrain}}/8$. As expected, the best results are achieved with the largest grid, i.e., (32×32) in the matching case. With the smallest grid, i.e., (16×16), the degradation is particularly significant for the lowest carrier-to-noise ratios. Moreover, in all the plots, the main factor of degradation is the decrease in the number of frequency steps $N_{f}$.

**Figure 7:**
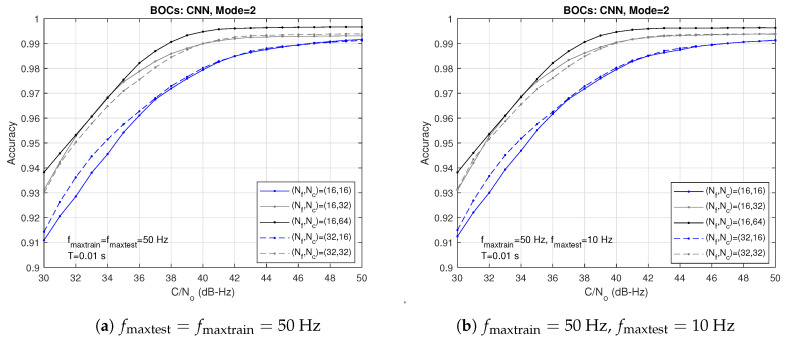
Accuracies obtained with correlators grids of different sizes $\left(N_{f},N_{c}\right)$ with $f_{\mathrm{maxtrain}}=50$ Hz.

Figure 7 exhibits the accuracies provided by correlators grids of different sizes $\left(N_{f}\times N_{c}\right)$, but now the NN has been trained with $f_{\mathrm{maxtrain}}=50$ Hz. Notice the striking contrast between the results of Figure 6 and Figure 7. In the latter case, the performance is almost independent of the number of correlators in the Doppler frequency ($N_{f}$), depending mainly on $N_{c}$, that is, the number of correlators in the code delay. As a result, Figure 7a,b, generated, respectively, with frequency matching and mismatch, are practically equal.

The receiver model considered in Figure 1 includes a grid of correlators that use information contained in the time domain (code delay) and frequency domain (Doppler frequency). This architecture was previously used, for instance, in [11] and further referred to in [17]. However, the novelty of our approach is characterized by the following aspects: (i) we developed mathematical tools to analytically define the additive noise components in each correlator, including their cross-correlations, (ii) we implemented models for multipath, during the training of the neural network, based on the DLR model, and (iii) we used DFT pre-processing of the correlators outputs to improve the CNN performance.

### 4.2. Multi-Observation Decisions

When the product $(C/N_{0})T$ is small, the probabilities $P_{fa}$ and/or $P_{md}$ tend to be high, leading to a significant decrease in the accuracy. This drawback is minimized by making decisions based on multiple observations of the grid of correlators in *N* adjacent intervals of duration *T*.

With hard tentative multipath/no multipath decisions generated by the NN in *N* consecutive correlation intervals (observations), we may apply the following criterion: select class $H_{1}$ only when there are, at least, *M* positive tentative decisions, with 1≤M≤N (M-of-N selection). We refer to a hard decision regarding this methodology based on the M-of-N selection. Let the probabilities of false alarm and missed detection for each tentative decision be, respectively, $P_{fa}$ and $P_{md}=1-P_{d}$, as defined in (36). The overall probability of false alarm (under hypothesis $H_{0}$) is

(40) P˜fa=∑n=MNNnPfan(1−Pfa)N−n

and the overall probability of correct decision (under hypothesis $H_{1}$) is

(41) P˜d=∑n=MNNnPdn(1−Pd)N−n.

Hence, the overall probability of missed detection is provided by ${\tilde{P}}_{md}=1-{\tilde{P}}_{d}$.

**Figure 8:**
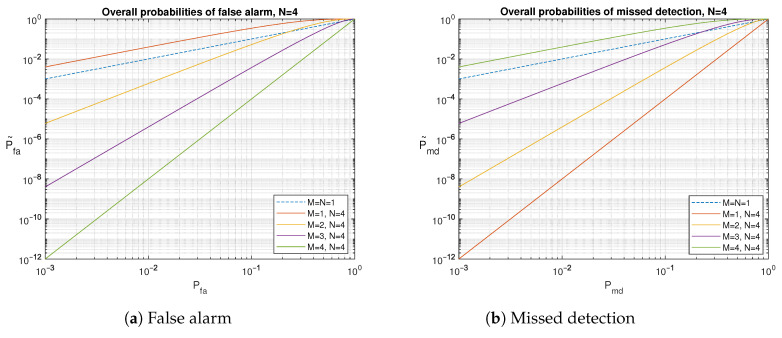
Example of overall probabilities of false alarm and missed detection for N=4.

Ideally, for multiple observations (N>1), the overall probabilities of false alarm and missed detection should decrease simultaneously relative to the corresponding probabilities of a single observation (N=1). However, this is achievable only for certain values of $P_{fa}$, $P_{md}$ and *M*, as illustrated in Figure 8 for N=4. In general, for a given value of $P_{fa}$ and *N* being constant, ${\tilde{P}}_{fa}$ decreases when *M* grows, and, for a fixed value of $P_{md}$ and *N* being constant, ${\tilde{P}}_{md}$ increases when *M* grows.

When *N* softmax values $s_{0}^{\left(n\right)}$ and $s_{1}^{\left(n\right)}$, n=1,…,N, are available in consecutive correlation intervals, we may add them before producing a final decision, according to

(42) $$S_{0}=\frac{1}{N}\sum_{n=1}^{N}s_{0}^{\left(n\right)},\;S_{1}=\frac{1}{N}\sum_{n=1}^{N}s_{1}^{\left(n\right)}$$

and deciding in favor of hypothesis $H_{0}$, if $S_{0}>S_{1}$, or $H_{1}$ otherwise. We refer to this as the soft decision to this methodology, in contrast to the alternative hard decision.

**Figure 9:**
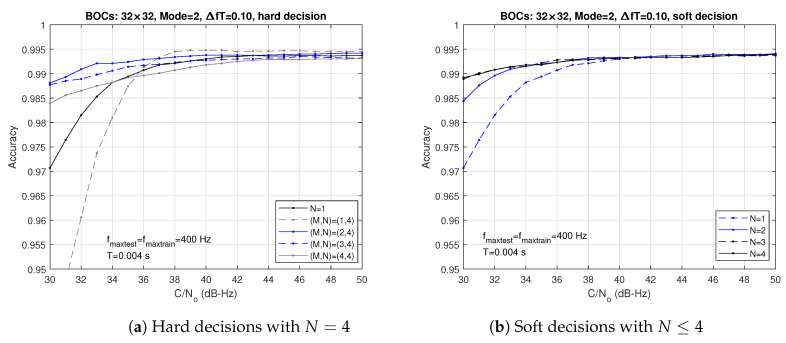
Accuracies obtained with multi-observation decisions and T=4 ms.

Figure 9 depicts the accuracies obtained with multiple observations using hard and soft decisions. The correlation interval is T=4 ms, which means that the classifier produces a decision every NT=4N ms. For the hard decision case, N=4 correlation intervals were used. Note that, for N=4, the best results achieved in both plots are approximately equivalent. However, using soft decisions, the accuracy is practically independent of *N*, provided that N≥3. As a consequence, soft decisions are preferable to hard decisions (at least for this example).

### 4.3. Multipath Mitigation Technique Using Soft Decisions

**Figure 10:**
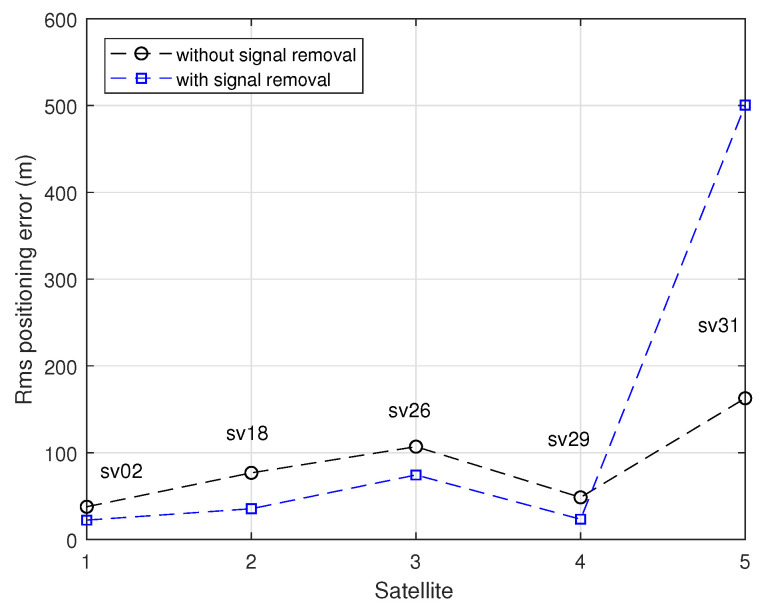
3D positioning errors obtained in the presence of one multipath-disturbed signal when the corresponding pseudorange measurement is either used (circles) or is removed from the navigation equation (squares).

With the goal of understanding the motivation for having an algorithm that detects the effect of multipath in a given received signal, consider the scenario described in Figure 10. The figure displays the three-dimensional (3D) positioning errors obtained with five GPS satellites in view (SV2, SV18, SV26, SV29, and SV31) when one of the received signals is affected by multipath, provoking an increment of 50 m in the corresponding pseudorange. In the absence of multipath, the rms positioning error is 22.0 m. This value includes the effect of thermal noise (it is assumed that the pseudoranges are disturbed by independent zero-mean Gaussian noises with standard deviation equal to 5 m). The circles indicate the rms positioning errors achieved when the pseudorange associated with multipath is included in the least-squares navigation solution (single-point solution, [33]). The different errors depend on the position of the corresponding satellite through the geometric dilution of precision (GDOP). The squares indicate the resulting errors when the pseudorange measurement of the multipath-disturbed signal is removed from the solution (strategy I).

The figure shows that, in most scenarios, if reliable information is available concerning the presence of multipath in a certain signal, the removal of that signal from the equation of navigation permits to reduce the resulting positioning error (although at the cost of a slight GDOP increase). However, when the SV31 signal is affected by a multipath effect and is removed from the equation of navigation, a substantial increment of the positioning errors is obtained. The explanation for this anomalous behavior is the very large growth in the GDOP that results from discarding SV31. This example enables concluding that strategy I (signal removal) may not be the best one if it leads to the significant growth of the GDOP parameter. We will see next that an alternative strategy (strategy II), based on the outputs of the NN, may provide a better multipath mitigation performance.

In fact, the availability of softmax values $S_{k}$ permits to devise new strategies to mitigate the multipath effect on the position, velocity, and time (PVT) solution as an alternative to removing the affected signal(s) from the equation of navigation. As the degree of confidence in the classification provided by the NN increases with the difference $|S_{1}-S_{0}|$, a weighted least-squares algorithm (or an extended Kalman filter having the observations noise covariance matrix with adjusted inputs) that uses *K* signals (with K≥5) may provide a more accurate estimate of the position, velocity, and time (PVT) than the least-squares solution based on K−1 signals. This strategy is particularly useful when a reduced number of visible satellites are available because the removal of one or more signals from the equation of navigation could significantly increase the GDOP or even prevent the computation of the PVT solution.

Suppose K≥4 satellites are being tracked. The pseudorange measurements, after correction of the ionospheric/tropospheric delays, are provided by [3]

(43) $$r_{i}=\sqrt{{\left(X_{i}-x\right)}^{2}+{\left(Y_{i}-y\right)}^{2}+{\left(Z_{i}-z\right)}^{2}}+\Delta_{r}c,\;i=1,\ldots ,K,$$

where $\left(X_{i},Y_{i},Z_{i}\right)$ is the satellite position, (x,y,z) is the receiver position, and $\Delta_{r}c$ is the range bias due to the receiver clock offset $\Delta_{r}$.

A least-squares solution $\left(\hat{x},\hat{y},\hat{z},{\hat{\Delta }}_{r}c\right)$ is difficult to obtain analytically for the set of pseudorange measurements (43) because the measurements are nonlinear, but the equations may be linearized by performing a Taylor expansion regarding the predicted user position and range bias $\left(\tilde{x},\tilde{y},\tilde{z},{\tilde{\Delta }}_{r}c\right)$. The single-point navigation solution using the weighted least-squares estimate is [34]

(44) $$\left[\begin{array}{c} \hat{x}\\ \hat{y}\\ \hat{z}\\ {\hat{\Delta }}_{r}c \end{array}\right]=\left[\begin{array}{c} \tilde{x}\\ \tilde{y}\\ \tilde{z}\\ {\tilde{\Delta }}_{r}c \end{array}\right]+{\left(G^{T}WG\right)}^{-1}G^{T}W\left[\begin{array}{c} r_{1}-{\tilde{r}}_{1}\\ r_{2}-{\tilde{r}}_{2}\\ \vdots \\ r_{K}-{\tilde{r}}_{K} \end{array}\right],$$

where *G* is the geometry matrix, *W* is the weighting matrix, assumed to be positive definite and symmetric, and ${\tilde{r}}_{i}$ is the predicted pseudorange. Let $\Lambda_{i}=S_{1,i}-S_{0,i}=1-2S_{0,i}$, i=1,…,K, denote the difference of the two softmax outputs for satellite *i*, with $0\leq S_{0,i}\leq 1$, where $\Lambda_{i}=-1$ indicates a no-multipath scenario with probability one and $\Lambda_{i}=1$ a multipath scenario with probability one. In contrast, $\Lambda_{i}=0$ corresponds to a minimum of confidence in the NN decision. Therefore, we propose the following (K×K) weighting matrix

(45) $$W=\left[\begin{array}{cccc} {\left(1-\Lambda_{1}\right)}^{\gamma } & 0 & \ldots & 0\\ 0 & {\left(1-\Lambda_{2}\right)}^{\gamma } & \ldots & 0\\ \vdots & \vdots & \ddots & \vdots \\ 0 & 0 & \ldots & {\left(1-\Lambda_{K}\right)}^{\gamma } \end{array}\right]=2^{\gamma }\left[\begin{array}{cccc} S_{0,1}^{\gamma } & 0 & \ldots & 0\\ 0 & S_{0,2}^{\gamma } & \ldots & 0\\ \vdots & \vdots & \ddots & \vdots \\ 0 & 0 & \ldots & S_{0,K}^{\gamma } \end{array}\right]$$

where γ (with γ≥0) is a selectable parameter that enables adjusting the range of values of the weights in *W*. For instance, conducting γ=0, the estimated position corresponds to the conventional least-squares solution obtained with *K* satellites because *W* is the identity matrix. The γ→∞ estimated solution is tantamount to the least-squares solution obtained with K−1 satellites (the signal affected by multipath aspects is discarded) as ${\left(1-\Lambda_{i}\right)}^{\gamma }\to \infty$, if $S_{1,i}<S_{0,i}$, and ${\left(1-\Lambda_{i}\right)}^{\gamma }\to 0$, if $S_{1,i}>S_{0,i}$. For the remaining values of γ, a weighted least-squares solution with *K* satellites is computed.

**Figure 11:**
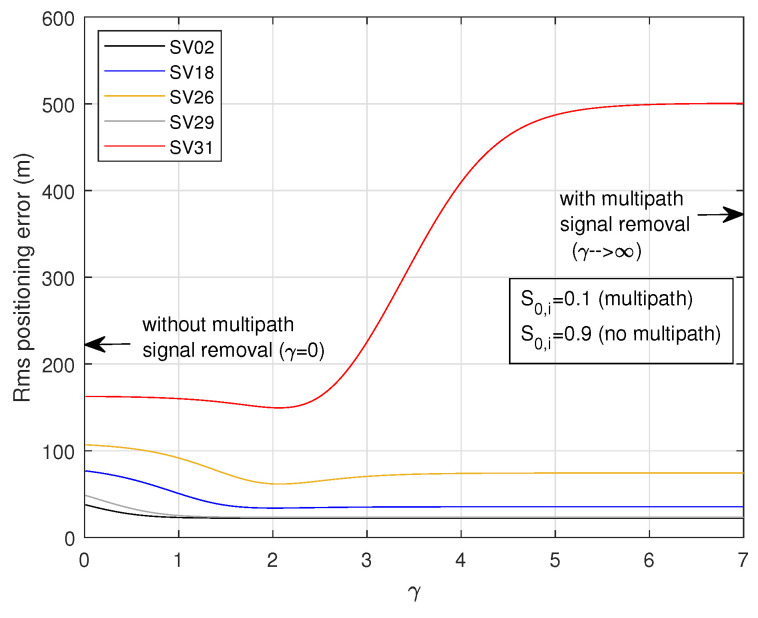
3D positioning errors obtained with one multipath-disturbed signal using softmax values and weighted least-squares solutions with different values of γ.

Figure 11 displays the rms 3D positioning errors versus γ for the same scenario described in Figure 10, provided by (44) and (45). In this simulation, we assumed that $S_{0,i}=0.9$ for satellite signals free of multipath and $S_{0,i}=0.1$ for satellite signals affected by multipath. Figure 11 shows that, when the signals of satellites SV02, SV18, SV26, or SV29 are affected by multipath aspects, the best solution consists of removing that signal from the equation of navigation (strategy I), but this strategy leads to poor results when the disturbed signal belongs to SV31. Overall, strategy II, which consists of using (44) with γ≈2, provides the best results in terms of multipath mitigation.

If, instead, an extended Kalman filter was employed, the weighting matrix *W* could be replaced by the covariance matrix, $R_{n}+R_{m}$, in the measurement (observations) model [35], with $R_{n}$ depending on the noise pseudorange terms and $R_{m}\propto W^{-1}$ depending on the multipath effect.

## 5. Conclusions

In this work, we proposed algorithms for the detection of GNSS signals affected by multipath aspects based on two neural network architectures: the multilayer perceptron (MLP) and the convolutional neural network (CNN). Extensive simulations have shown that the CNN is, in general, superior to the MLP, thus becoming the adopted solution. To obtain a classifier that is robust to various types of multipath aspects, the network was trained with synthetic noisy signals generated from a blend of different multipath scenarios that characterize the DLR model. The testing of the neural networks was performed with a set of multipath scenarios affected or not by the multipath effect with equal probabilities. No specific model was adopted for the testing task in order to make it as general as possible.

In the GNSS receiver, the neural network inputs were produced by a grid of $N_{f}N_{c}$ correlators that covered the Doppler frequencies and the code delays of the different received replicas. The number of correlators has a major impact on the computational effort and should be kept as small as possible. However, decreasing the number of correlators, in particular those in the frequency domain ($N_{f}$), tends to negatively affect the performance of the classifier. Good results were obtained for the Doppler range of ±400 Hz by conducting $N_{f}=32$ and $N_{c}=16$.

Since the neural network was trained using Monte Carlo techniques, increased emphasis was placed on the analytic characterization of the cross-correlations between the noise components in the different correlators. It was also found that pre-processing the CNN inputs with a 2D Discrete Fourier Transform enabled significantly improving the detection performance in certain scenarios. The performance achieved with the CNN was compared with the MLP, which was used as a benchmark. In general, the CNN provided better results than the MLP, especially in low-signal-to-noise-ratio conditions. Thus, the CNN was used as the main multipath detection algorithm in the current work. The explanation for the better performance of the CNN compared to the MLP architecture is that the former includes an initial part (convolutional and max-pooling layers) to perform automatic feature extraction before carrying out the classification task. In fact, the CNN may be viewed as a feature extractor followed by a classifier.

Depending on the type of the CNN outputs, hard or soft decisions could be utilized. When hard decisions are used, the GNSS signal affected by the multipath aspects is removed from the equation of navigation. The adoption of soft decisions enables implementing an alternative strategy for multipath mitigation, which consists of solving the equation of navigation using a weighted least-squares algorithm (or an extended Kalman filter), with the processing of each GNSS signal being affected by a different weight (or probability) provided by the neural network. The result is a decrease in the receiver’s position, velocity, and timing errors while keeping the number of processed signals unchanged, which may be a significant advantage when the presence of visible satellites has already been reduced.

## Data Availability

Data are contained within the article.

## Appendix A. Tables of DLR Model Parameters

The following tables were obtained using data from [20]. The second column of Table A1 indicates the shadowing probabilities.

**Table A1:** Parameters for the direct path.

Environment	Shad. Prob.	ρ (dB) [No Shad]	μ (dB) [Shad]	σ (dB) [Shad]
open, 25°	0	6.0	-	-
$45^{\circ }$	0	10.4	-	-
rural, $25^{\circ }$	0.96	10.8	−9.9	3.3
$45^{\circ }$	0.79	4.7	−5.4	2.3
suburban, $25^{\circ }$	0.59	4.7	−6.0	3.5
$45^{\circ }$	0.43	4.0	−7.2	3.2
urban, $25^{\circ }$	0.79	3.2	−12.1	6.3
$45^{\circ }$	0.56	8.5	−3.0	2.7
highway, $25^{\circ }$	0.19	8.4	−5.8	1.7
$45^{\circ }$	0	7.8	-	-

**Table A2:** Parameters for near echoes.

Environment	λ	$\tau_{e}$ (ns)	*b* (μs)	$S_{0}$ (dB)	*d* (dB/μs)
open, $25^{\circ }$	1.2	400	0.03	−28.6	1.0
$45^{\circ }$	0.5	400	0.027	−29.0	1.1
rural, $25^{\circ }$	1.5	400	0.055	−24.9	19.2
$45^{\circ }$	1.8	400	0.051	−24.5	13.4
suburban, $25^{\circ }$	1.4	400	0.038	−23.8	23.7
$45^{\circ }$	1.5	400	0.027	−24.4	23.0
urban, $25^{\circ }$	4.0	600	0.063	−17.0	26.2
$45^{\circ }$	3.6	600	0.081	−23.5	8.5
highway, $25^{\circ }$	2.2	600	0.077	−25.8	7.3
$45^{\circ }$	1.8	600	0.043	−27.1	29.5

**Table A3:** Parameters for far echoes.

Environment	λ	$2\sigma^{2}$ (dB)	$\tau_{\max}$ (μs)
flat terrain, $25^{\circ }$	0.3	−26.4	15
$45^{\circ }$	-	-	15
rural, $25^{\circ }$	0.8	−28.2	5
$45^{\circ }$	-	-	5
hilly, $25^{\circ }$	1.2	−29.0	10
$45^{\circ }$	-	-	10
mountainous, $25^{\circ }$	1.8	−28.5	15
$45^{\circ }$	4.0	−21.7	15

## Appendix B. Generation of a Gaussian Vector with a Given Covariance Matrix

Consider the generation of a generic Gaussian noise vector $U={\left[u_{1},\ldots ,u_{M}\right]}^{T}$, with zero mean and covariance matrix C, from the noise vector $W={\left[w_{1},\ldots ,w_{M}\right]}^{T}$ of zero-mean independent Gaussian components with unity variance. Let U=GW, where G(M×M) is a square matrix. The problem consists of determining *G* from $C=GE\left\{WW^{T}\right\}G^{T}=GG^{T}$. According to the spectral theorem for real symmetric matrices [36], the covariance matrix can be written in terms of its eigenvalues and eigenvectors as $C=V\Lambda V^{T}$, where *V* is an orthogonal matrix whose columns are the orthonormal eigenvectors of C and $\Lambda =\mathrm{diag}\;\{\lambda_{1},\lambda_{2},\ldots ,\lambda_{M}\}$ is a diagonal matrix with the real nonnegative eigenvalues of C. This leads to $G=V\Lambda^{1/2}=V\mathrm{diag}\;\left\{\sqrt{\lambda_{1}},\;{\sqrt{\lambda }}_{2},\ldots ,\sqrt{\lambda_{M}}\right\}$.
